# Automation and Standardization—A Coupled Approach towards Reproducible Sample Preparation Protocols for Nanomaterial Analysis

**DOI:** 10.3390/molecules27030985

**Published:** 2022-02-01

**Authors:** Jörg Radnik, Vasile-Dan Hodoroaba, Harald Jungnickel, Jutta Tentschert, Andreas Luch, Vanessa Sogne, Florian Meier, Loïc Burr, David Schmid, Christoph Schlager, Tae Hyun Yoon, Ruud Peters, Sophie M. Briffa, Eugenia Valsami-Jones

**Affiliations:** 1 Division 6.1, Federal Institute for Material Testing and Research (BAM), Unter den Eichen 44-46, 12203 Berlin, Germany; dan.hodoroaba@bam.de; 2 Department of Chemical & Product Safety, German Federal Institute for Risk Assessment, Max-Dohrn-Strasse 8-10, 10589 Berlin, Germany; harald.jungnickel@bfr.bund.de (H.J.); jutta.tentschert@bfr.bund.de (J.T.); andreas.luch@bfr.bund.de (A.L.); 3 Postnova Analytics GmbH, Rankine-Strasse 1, 86899 Landsberg, Germany; vanessa.sogne@postnova.com (V.S.); florian.meier@postnova.com (F.M.); 4 Centre Suisse d’Electronique et de Microtechnique (CSEM), Bahnhofstrasse 1, 7302 Landquart, Switzerland; loic.burr@csem.ch (L.B.); david.schmid@csem.ch (D.S.); 5 Vitrocell Systems GmbH, Fabrik Sonntag 3, 78193 Waldkirch, Germany; c.schlager@vitrocell.com; 6 Department of Chemistry, College of Natural Sciences, Hanyang University, Seoul 04763, Korea; taeyoon@hanyang.ac.kr; 7 Institute of Next Generation Material Design, Hanyang University, Seoul 04673, Korea; 8 Wageningen Food Safety Research, Wageningen University & Research, Akkermaalsbos 2, 6708 WB Wageningen, The Netherlands; ruudj.peters@wur.nl; 9 School of Geography, Earth and Environmental Sciences, University of Birmingham, Birmingham B15 2TT, UK; s.m.briffa@bham.ac.uk (S.M.B.); e.valsamijones@bham.ac.uk (E.V.-J.)

**Keywords:** sample preparation, automation, nanomaterial analysis, standardization

## Abstract

Whereas the characterization of nanomaterials using different analytical techniques is often highly automated and standardized, the sample preparation that precedes it causes a bottleneck in nanomaterial analysis as it is performed manually. Usually, this pretreatment depends on the skills and experience of the analysts. Furthermore, adequate reporting of the sample preparation is often missing. In this overview, some solutions for techniques widely used in nano-analytics to overcome this problem are discussed. Two examples of sample preparation optimization by automation are presented, which demonstrate that this approach is leading to increased analytical confidence. Our first example is motivated by the need to exclude human bias and focuses on the development of automation in sample introduction. To this end, a robotic system has been developed, which can prepare stable and homogeneous nanomaterial suspensions amenable to a variety of well-established analytical methods, such as dynamic light scattering (DLS), small-angle X-ray scattering (SAXS), field-flow fractionation (FFF) or single-particle inductively coupled mass spectrometry (sp-ICP-MS). Our second example addresses biological samples, such as cells exposed to nanomaterials, which are still challenging for reliable analysis. An air–liquid interface has been developed for the exposure of biological samples to nanomaterial-containing aerosols. The system exposes transmission electron microscopy (TEM) grids under reproducible conditions, whilst also allowing characterization of aerosol composition with mass spectrometry. Such an approach enables correlative measurements combining biological with physicochemical analysis. These case studies demonstrate that standardization and automation of sample preparation setups, combined with appropriate measurement processes and data reduction are crucial steps towards more reliable and reproducible data.

## 1. Introduction

Automation in analytical chemistry is an old subject that has been the subject of debate for several decades [1]. However, in the coming years, a paradigm change is expected based on recent analytical trends [2]. More specifically, there is currently a trend for all fields relevant to the development of new chemicals and materials to be revolutionized: Preparation, characterization, data management and analysis, and design of experiments. Today, preparation is handled by experimentalists and depends on their knowledge and experience. Robot-based synthesis and flow chemistry are becoming increasingly important, and this will lead to further automation, parallelization, and miniaturization of the sample preparation. Furthermore, automated systems allow the handling of larger batches and quantities. They are thus important for the upscaling of procedures from the lab to the industrial scale.

**Figure 1:**
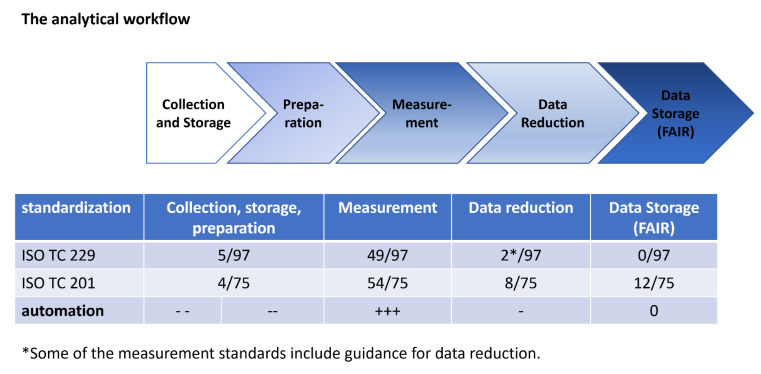
The analytical workflow consists of four steps: (i) collection and storage, (ii) preparation, (iii) measurement and (iv) data reduction. The procedures presented in this review are focused on sample preparation (i.e., stage ii). The number of standards concerning the different steps of the analytical workflow is given as a proportion of all published standards in the two technical committees 229 “nanotechnologies” and 201 “surface analytics”. The total number of standards contain non-technical standards, e.g., for terminology or toxicological testing which do not consider the analytical workflow. The degree of automation shown is arbitrary and ranges from low (---) to high (+++).

Nowadays, the characterization itself, i.e., the recording of analytical data, is highly automated, especially for physicochemical analysis methods such as spectroscopy, microscopy, diffraction, and scattering. Due to the developments in chemometrics, data analysis has also become increasingly automated in recent years [3]. Artificial intelligence will lead to further automation in data analysis, even in areas that have traditionally required more manual examination such as image analysis in microscopy [4]. In addition to increased efficiency, this trend is also expected to enhance reproducibility [2]. Measurement and data reduction can be saved as metadata of the results and reproduced consistently with the FAIR (findable, accessible, interoperable, and reproducible) guiding principles for scientific data [5]. The appropriate documentation of the experimental conditions in metadata will lead to a quantum leap in the reproducibility of experiments. In this development of automation, one particular step is rarely considered in the characterization process of materials. This is the sample preparation or the pre-analysis treatment. In the same way as for material synthesis, this step is usually performed manually by analysts. It must be ensured that the sample is not modified during its preparation. The traceability of this step is crucial for a deeper understanding of the characterization results. A prominent example of the importance of careful preparation methods is those associated with nanomaterials. These have been gaining increasing attention in recent years for both industrial applications and consumer products, in areas as diverse as catalysis, refinery, electronics, food, packaging, cosmetics, pharmaceutical, and medical devices [6,7,8,9]. Due to the increasing importance of these materials as well as the enormous influence of their properties (e.g., particle size) on their behavior, reliable analysis of these properties is a requirement for research, toxicology, labeling, and classification purposes as well as their acceptance in the society. Previous work has shown the degree to which storage conditions can influence the properties of nanoparticles over time. In addition to possible alteration of particle properties due to unsuitable storage, rigorous published studies have shown that inconsistent sample preparation procedures are a critical source of variability in the characterization of nanomaterials [10,11]. In addition to the potential changes in nanomaterials due to environmental influences (resulting from properties such as their large surface area), factors such as different preparation methods and variations within those methods can dramatically influence analytical results. For example, different analytical techniques and analytical questions require different manifestations of the sample, such as powder, suspension, aerosol, prepared on a substrate, or embedded in another material. In addition, not every preparation protocol is suitable for every material or analytical question. All these challenges are important as this step is the first in the analytical chain after the sample collection and storage (Figure 1). This means that errors in the preparation propagate in the following steps. In other words, precise and well-defined measurements and data reduction cannot redress errors in the sample preparation. Therefore, more attention must be paid to this step when discussing the reproducibility and reliability of the analytical results.

The aim of this publication is to discuss how automation can facilitate reaching the goal of better transparency and, at the same time, reproducibility in sample preparation prior to measurement.

As mentioned earlier, the third step in the analytical workflow, the measurement performed on the sample, is in many ways automated nowadays, due to the increasing sophistication and user-friendliness of most analytical instruments. For several methods, this process is either already standardized or in the progress [12]. As shown in Figure 1, most standards in ISO committees representative of nano-analysis are related to the measurement, whereas for the preparation steps only a few standards exist. It must be noted that for every standardized method, a scientific consensus is firstly established on how to perform such measurements, usually via national or international norm committees and/or using methods such as inter-laboratory comparisons. There is an ongoing discussion about the optimal standardized data format and the necessary contents of metadata describing all relevant parameters for nanomaterial analysis concerning production, collection, sample preparation, and finally measurement. This is reflected in the FAIR (findable, accessible, interoperable, reusable) data initiative which, amongst other priorities, aims to reduce reproducibility issues around nanomaterial analysis by promoting the collection, storage, and availability of analytical metadata [2]. The next steps towards automation are taking place in data reduction, i.e., post-measurement data analysis, where developments in chemometrics and artificial intelligence are leading to a high degree of automation for this process. Once again, a comprehensive description of the procedure is required to allow accurate reproduction of the experiment. The best way to enable reproducibility is the use of metadata for the measurement process. Even for “traditional” manual data reduction, the quality of the data reduction and interpretation can be evaluated and peer-reviewed if the raw data and the metadata are shown in detail. In recent years, efforts have been initiated to establish guidelines or standards.

In contrast, the automation of the first steps of the analytical chain, sample collection, storage, and preparation, has not attracted a similar amount of attention, although incorrect handling can lead to irreversible changes of the nanoparticles. Usually, nanoparticles are prepared in liquid suspension and are available as such. In this preparation, the samples can be characterized with different methods (Table 1). Most of these methods, e.g., DLS, SAXS, or PTA (particle tracking analysis), give some information about the particle size. Other methods such as chromatography, fractionation, or electrophoresis allow for the separation of the nanoparticles in the suspension depending on specified properties such as particle size or surface modifications. Additionally, the detection of the measurand is relatively fast, in the time range of seconds or a few minutes, which allows the application of these methods for quality control in the production of nanoparticles to track deviations from the desired properties. For this aim, in situ or in operando techniques were developed. These allow direct measurements on a production line [13]. Another exciting development in recent years is robot platforms which allow autonomous high-throughput experiments. Such automated equipment can be used for the optimization of experimental conditions, e.g., concentration or temperature range [14], which require a lot of time and manpower when traditional manual approaches are used.

The following tables give an overview of characterization methods and suitable preparation methods. Hereby, the tables are ordered for different preparation methods. No claim of completeness is made, but well-established and commonly used preparation methods and characterization techniques have been listed. It is recommended that the experimentalist assesses thoroughly the suitability of analytical and preparation methods to be used on the basis of the sample properties to be measured. Thereby, the limits or challenges of each method must be considered, such as appropriate concentration ranges or possible alteration of sensitive nanoparticles due to the preparation or the analytical method.

A major drawback when dealing with suspensions is that samples in this form are not suited to surface analytical or microscopic methods, which require (ultra-) high vacuum, e.g., X-ray photoelectron spectroscopy (XPS), secondary ion mass spectrometry (SIMS), scanning and transmission electron microscopy (SEM and TEM) (Table 2), unless they are dried onto an appropriate substrate. On the other hand, the surface analysis methods allow insight into the surface chemistry of the nanoparticle which cannot be obtained by means of other methods. Furthermore, microscopic methods, able to provide, e.g., the shape of individual particles, are complementary to the scattering methods as ensemble methods. Therefore, they are needed for the validation of the scattering methods used in quality control. The preparation of nanoparticles for these techniques to ensure reliable measurements is the subject of ongoing discussion in the literature [11,15]. Some recommendations are discussed later.

Nanomaterials are not only provided as suspensions but also often as powders. Such powders can be transferred into liquid and thus handled as suspensions. However, occasionally, it is not possible, or it is extremely complex to prepare a homogeneous and stable suspension from a powder without causing changes to sensitive nanomaterials. Therefore, such preparation of suspensions from powders must be performed with great care. For this reason, reliable and reproducible methods to prepare powders are as important as those related to handling suspensions (Table 3). In principle, all analytical methods which can be used for dried suspensions are suitable for powders too. Notably, a small number of well-established analytical techniques can only work with dry powders; for example, the measurement of surface area with the Brunauer, Emmett, and Teller (BET) method and thermogravimetry to measure mass loss due to thermal treatment of nanomaterials. These methods are highly informative provided the sample quantity is sufficient. Both methods require no elaborate preparation and are often used as a first overview of the sample. Therefore, they are usually applied for quality control. The great advantage of the measurement of samples as powders is that they can be measured as received. On the other hand, fixation cannot always be ensured, and nanoparticles can even potentially contaminate the whole experimental equipment if not properly attached to their substrate. This can lead to cross-contamination and damages of the often very expensive equipment used for surface analysis or electron microscopy. Powders are a good example of a preparation method that is not suitable for automation because it is relatively simple. On the other hand, the inspection of the fixation requires the critical look of the experimentalist and cannot be replaced by automated systems.

To overcome this latter problem, powders can often be pressed into pellets thus creating a solid surface for characterization (Table 4). However, during preparation, the sample can be contaminated, or the surface can be damaged. To overcome such problems, plasma treatment or sputtering with ions or charged clusters for cleaning the surface of the pellet is possible.

In recent years, some more sophisticated sample preparation methods have been developed (Table 5). Cryo treatments, such as cryo-fixation or freeze-drying, are methods that are used for sensitive samples, especially organic or biological samples. With cryo-fixation, not only the nanoparticles can be maintained but their surrounding organic matrix can also be maintained. This is important for investigating the nanoparticles in biological media, such as cells [16]. Due to the experimental experience required for these methods, the automation is rather complex and currently not recommended.

For microscopy and other methods allowing the analysis of single particles, some promising techniques have recently been developed (see Supplementary Materials Figures S1 and S2). Microprinting devices are a promising possibility for automating the preparation [17]. This approach is an important step for using imaging methods as standard methods and is not only restricted to electron microscopy. Other imaging methods such as scanning Auger mapping or ToF-SIMS can be used. However, several challenges remain for its broad application. Further optimization of the particle concentration in suspension and optimization of the droplet drying process on the substrate are necessary to avoid agglomerates and coffee rings. In addition, improvements in the alignment technique using computer-assisted detection of alignment markers would increase the precision and speed of printing. Further improvements would involve the printing of even smaller droplets, which in turn would enable faster imaging of entire drops and the printing of 1 drop per TEM grid cell leading to more than 100 different samples on one single TEM grid. 

An alternative to this microprinting approach was demonstrated to be electrospray deposition which relies on the strong electrostatic repulsion forces induced by a corresponding electrical field of several kV in which a liquid suspension droplet of nanoparticles is injected [18,19]. Another promising method of nanoparticle sample preparation with high representativity for imaging techniques is the cross-sectional sample preparation method. Appropriate embedding of a powder particulate sample into a resin matrix followed by fine grinding and polishing, and eventually an ultrathin conductive coating applied in an optimized sequence is reported to work successfully for routine, automated nanoparticle analysis by SEM [20,21]. Once an optimally dense dispersion of the particles in the resin is obtained, together with a high-quality surface polishing, a very large number of nanoparticles can be measured automatically and thus the representativity of the particle size measurement is achieved. For this sophisticated experimental approach, an automated routine is highly desirable and an important step to a fully automated analysis. Alternatively, the particles can be fixed on a polymer matrix [22].

Standardization is an elaborate process that is driven by national and international standardization bodies. Usually, standardization is triggered by commercial or industrial interests to instill trust in the supply chains [23]. Therefore, it is perceived as a broad consensus approach, involving well-established steps to obtain reliable and reproducible results. In the process of standardization, reliability and reproducibility are usually verified by suitable interlaboratory comparisons. In Table 6, some examples of international standards of sample preparation methods are listed. Several projects dedicated to specific nanomaterials or methods are in progress.

Initial efforts towards standardization, particularly for preparation, have been achieved in the framework of the ACEnano project (https://cordis.europa.eu/project/id/720952 (accessed on 31 December 2021)) which was funded by the European Union with the aim to develop new reliable methods suitable for the analysis of nanomaterials. Other regional or national organizations such as the European Committee for Standardization (CEN) or the National Institute of Standards and Technologies (NIST) publish widely accepted standards on their websites [24], e.g., preparation of nanoparticles dispersions from powdered material using ultrasonic disruption [25]. All these standards provide reliable guidelines for sample preparation.

**Figure 2:**
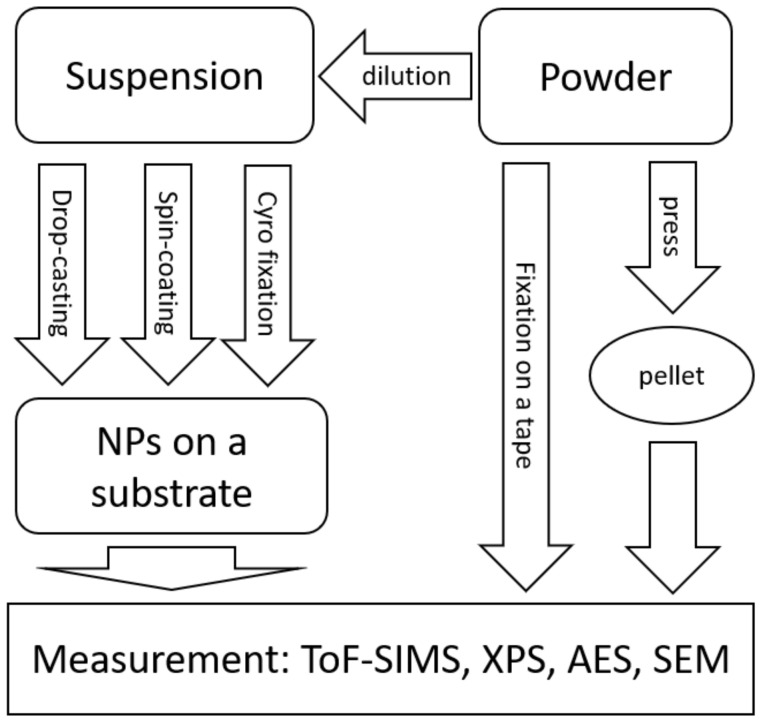
Overview of sample preparation techniques for surface analytical methods.

A good example of efforts in harmonizing and optimizing preparation protocols is the field of surface analytics. Surface analytical methods play a crucial role in the characterization of nano-objects, because the unique properties of nanoparticles arise substantially from their large specific surface area. For very fine nanoparticles, smaller than 5 nm, the surface dominates sample volume. Therefore, the integrity of the surface should not be influenced by sample preparation. Preparation methods such as drop-casting, spin-coating, preparation of powders and pellets, and even cryo-fixation which were described recently [26] should be adapted to each analytical task (see Figure 2). For surface analytical methods, the homogeneity of the sample must be ensured, and the influence of the substrate should be minimized. For typical surface analytical issues, a homogeneous closed monolayer is the most suitable preparation method, whereas for single particle analysis a capability to isolate individual particles is essential for analysis.

Central to ensuring reproducibility of sample preparation is the provision of a detailed process description including all relevant steps in a workflow; in other words, a standard operating procedure (SOP) [27]. Especially for scientific publications, an extensive step-wise description of the protocol should be a requirement. Special attention is needed to describe any possible sample alteration, e.g., during the transfer of powders to suspensions. This avoids the increased workload of optimizing preparation procedures developed separately by each group and leads to the dissemination of successful preparation protocols which become “standards” in the nanoscience community.

Two approaches in the field of automated sample preparation will be presented in the next section. The method selection was based on issues described earlier, i.e., the need for reliable and harmonized sample preparation methods that can address concerns regarding manual performance quality depending strongly on the experimentalists and their experience. The approach has thus been to introduce automation, which can boost the reliability and reproducibility of sample preparation, an issue also recently discussed in the context of nanomaterial synthesis [2]. The advantages of automation are, firstly, an increase in the number of experiments possible, thus also increasing the possibilities to obtain the best experimental conditions and the statistical relevance of the preparation method. Furthermore, each preparation is traceable, because the reaction conditions belong to the metadata of the analytics and can easily be read out as a protocol. Each new preparation under the same conditions should provide comparable results, and these should be reproducible by different labs. Differences in the characterization results can clearly be correlated with the sample itself and not with independent uncertainties in the sample preparation. Furthermore, the throughput of analysis can be increased and costs and working hours reduced. Hereinafter, the first approaches in the automation of the sample preparation will be described.

## 2. Results

In the ACEnano project, new methods were developed and existing methods were optimized for nano analysis. Within this project, several efforts were made to develop automated preparation systems, two examples of which are presented here.

Firstly, as discussed in the introduction, a lot of measurement techniques require samples in the form of stable and homogeneous suspensions. Therefore, a robot-based station is presented here. Analytical methods such as asymmetrical flow FFF coupled to multi-angle light scattering (AF4-MALS) can be integrated which allows a fully automated analytical workflow. The results of this automated system are compared with those from manual preparation procedures.

The second system allows finding a realistic and robust procedure for the exposure of biological systems, such as cell cultures, to nanomaterials. Such a preparation procedure is crucial to investigate bio-nano interactions. A system suitable for reproducible cell exposure to inhalable nanoparticles at the air–liquid interface is presented. With this system, it is possible to prepare nanoparticles for TEM investigations at the same time as the experimental exposure is taking place. This approach allows correlative measurement of the biological activity or the nanoparticles with their size and shape. Such investigations further allow establishing structure–activity relationships.

### 2.1. Robot-Based Station for the Automated Preparation of Nanomaterial Suspensions

The automated preparation of liquid samples containing small molecules is an integral part of many analytical workflows with commercial solutions already on the market enabling reliable and often high-throughput analyses [28]. However, despite the scientific community’s awareness of the human impact on reproducibility and reliability of measurement results in nanomaterial characterization especially in suspension, automation in this field is still underdeveloped [29,30].

**Figure 3:**
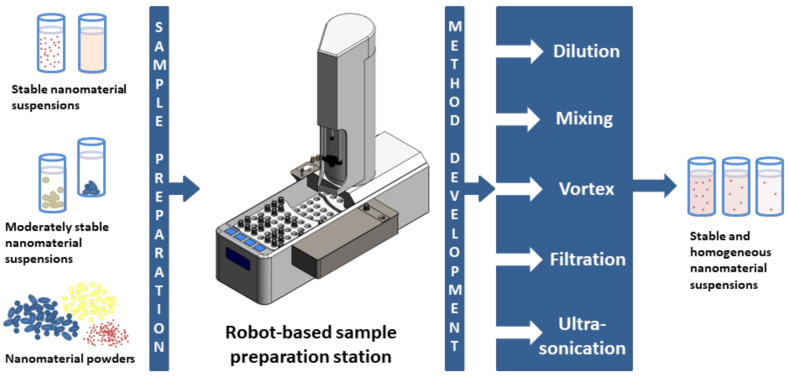
Schematic of the robot-based sample preparation station and its functionalities including the ultrasonication vial tweeter (dark grey box) developed and integrated as part of the ACEnano project.

For this reason, a robot-based station for the automated preparation of nanomaterial suspensions was developed within the ACEnano project. This station combines the functionalities already established in liquid chromatography applications (diluting, mixing, vortexing, and filtration) with the demands known from nanomaterial sample preparation. For this purpose, a dedicated ultrasonication device in the form of a vial tweeter was developed and integrated into the station enabling the application of ultrasound energy without direct contact of the ultrasonication probe with the nanomaterial sample itself (Figure 3).

The overall aim of this robot-based sample preparation station development was to provide a flexible, adjustable, and automated sample preparation platform that can address the complexity of the preparation of stable nanomaterial suspensions and that can also be integrated into an automated analytical workflow for example in combination with well-established nanomaterial characterization techniques such as, e.g., DLS or FFF.

A further important objective, especially from a commercial point of view, was to investigate the performance of the robot-based station compared with a manual sample preparation performed by experienced lab staff not only in terms of reproducibility and precision but also with respect to process duration. This was in-house validated against a series of three nanomaterial samples of different stability and complexity including two suspensions and a powder sample. Further information about the investigated nanomaterial samples is listed in Table 7.

In-house validation of the robot-based station was performed and tested against manual preparation using dedicated sample preparation protocols specifically developed and optimized for each respective nanomaterial sample. The applied procedures included “suspending”, “diluting”, “vortexing”, “mixing”, and “ultrasonication” and are summarized in Table 8. It is important to note that the manual sample preparation was performed in exactly the same manner in order to ensure the best comparability with the results obtained from the automated approach. Furthermore, sample preparation was performed on different days with six independent aliquots per nanomaterial sample in order to ensure a reliable calculation of the respective uncertainty budgets. The investigated experimental parameters included linearity of dilution expressed by the squared correlation coefficient R^2^ obtained from UV absorption analyses and particle size distribution after finalized preparation as z-average of the hydrodynamic diameter obtained from DLS measurements.

The results obtained from the dilution experiments (Table 9) indicate an excellent linearity of the squared correlation coefficient R^2^ with practically no difference between the manual and the automated dilution in terms of precision at least for the stable AuNP and the moderately stable TiO_2_-PVP sample. However, while still exhibiting very good linearity, an increased standard deviation for the automated in comparison to the manual preparation of the pyr. SiO_2_ sample can be observed. 

A similar trend is also visible for the particle sizes obtained for the TiO_2_-PVP and the pyr. SiO_2_ powder sample (Table 10). This demonstrates that the robot-based station can provide similar results in comparison to the manual sample preparation with a slightly better precision at least for the moderately stable TiO_2_-PVP sample. For the more challenging pyr. SiO_2_ powder sample, however, the automated preparation revealed a measurable lower precision including a significantly larger particle size in comparison to the manual preparation. These observations might be related to the challenges that come with obtaining a powder sample as a stable and homogeneous suspension. For example, enhanced sticking of the powder to the glass wall of the sample vial or increased sedimentation tendency due to larger particle sizes or larger agglomerates present in the sample, are effects that can be better compensated during a manual rather than an automated sample preparation. Furthermore, the observed deviations in the measured particle sizes might also be related to bias introduced by the DLS analysis, which is known to overestimate the overall particle size distribution in the presence of even only small amounts of larger particles or agglomerates [31].

To investigate whether the observed variances in the measured particle size are related to an inappropriate sample preparation procedure leading to an unstable final sample suspension or to the applied DLS analysis, an optimized sample preparation procedure for the pyr. SiO_2_ powder was developed (Table 11). To obtain a more reliable indication of the size distribution of such a polydisperse sample, instead of DLS, prepared samples were analyzed by asymmetrical flow FFF coupled to multi-angle light scattering (AF4-MALS). The latter is a high-resolution analytical technique particularly useful for the fractionation and characterization of such complex samples [31,32,33]. Injection of both manually and automatically prepared samples into the AF4-MALS system was thereby realized using the “Draw&Dispense” function of the robot-based station and a six-port injection valve equipped with a 100 µL sample loop that connected the station with the AF4-MALS system. Again, to ensure the best comparability of the results, both manual and automated sample preparation, as well as subsequent AF4-MALS analysis, were performed in the most similar way possible (conditions are given in Supplementary Materials Table S2 and Figure S3). The only difference that needs to be highlighted here is the fact, that in the case of the robot-based station, a completely automated analytical workflow from sample preparation to analysis could be established without the need for human involvement except for the initial gravimetrical step and final data evaluation.

**Figure 4:**
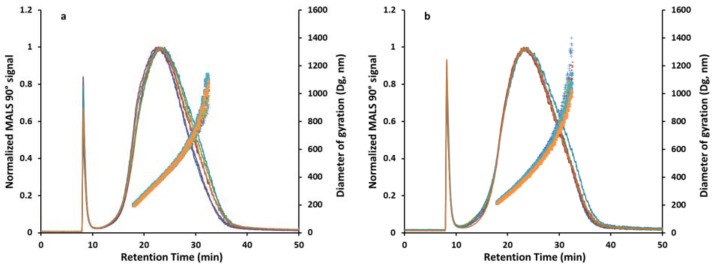
(**a**) Overlay of AF4-MALS (Postnova AF2000 MultiFlow AF4, Postnova PN3621 MALS) fractograms obtained from the analysis of six independent pyr. SiO_2_ samples which were prepared using *the robot-based system*. (**b**) Overlay of AF4-MALS fractograms obtained from the analysis of six independent pyr. SiO_2_ samples which were prepared *manually by experienced lab staff*. In all fractograms, the 90° MALS signal (line) is plotted against the diameter of gyration (dots) calculated from MALS angular data using the random coil model. All investigated samples were analyzed in singular (n = 1). The measurements of the six independent samples are presented in different colors.

Figure 4 displays the AF4-MALS fractograms that were obtained for six independently prepared pyr. SiO_2_ samples processed either automatically using the robot-based station (Figure 4a) or manually (Figure 4b). Key endpoints that were assessed to investigate the reproducibility of the sample preparation procedure and to also enable the direct comparison between both approaches included (i) the particle size displayed as the diameter of gyration (D_g_) at the maximum of the 90° MALS signal, (ii) the arithmetic mean of D_g_ across the elution window from 18–32.5 min as a representative of the particle size distribution across the eluting sample as well as (iii) the 90° MALS peak width displayed as the full width at half maximum (FWHM). The obtained findings are summarized in Table 12.

Firstly, it can be stated that the optimization of the sample preparation procedure resulted in a much higher stability of the obtained pyr. SiO_2_ suspensions displayed by the low standard deviations (<7%) observed for all three investigated AF4-MALS endpoints and independent from the respective sample preparation approach, i.e., automated or manual. This highlights that the initially observed low reproducibility of the DLS results (Table 10) is in fact relatable to an inappropriate sample preparation procedure leading to unstable suspensions rather than to the degree of automation of the sample preparation procedure or the performed DLS analysis. Moreover, the noticeably low standard deviations of the AF4-MALS results also indicate a comparably good reproducibility between the automated and the manual sample preparation approach with a slightly lower absolute particle size observed for the automated procedure, which might be related to very small differences in the manual handling of the sample vial. Especially after the last ultrasonication step, a slightly longer time gap between sample preparation and subsequent analysis, for example, may already lead to a colder sample that might eventually lead to a measurably increased agglomeration that is difficult to control.

**Figure 5:**
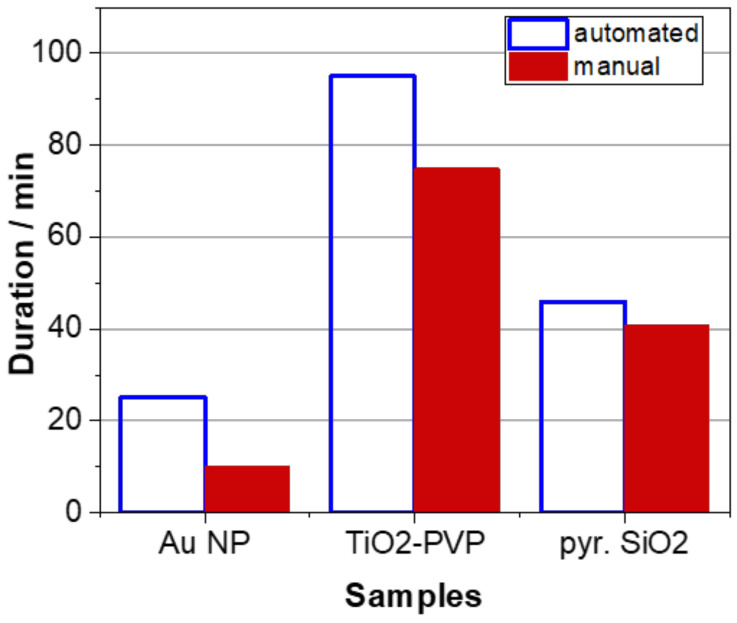
Comparison of the duration of the sample preparation procedures either performed automatically using the robot-based station or manually by an experienced lab user. Numbers represent the duration of the sample preparation following the procedures described in Table 8 and Table 11 for a single processed sample, respectively.

Taking into account the duration of the sample preparation, it can be concluded that experienced lab staff can process a nanomaterial sample generally faster than the robot-based station; however, this effect levels out increasingly with a higher complexity of the respective sample (Figure 5). In this respect, it must be emphasized that the applied automated procedure is highly flexible and can be easily adjusted to a minimum standard set by each user individually that may eventually contribute to an overall significantly reduced sample preparation time while even improving its reproducibility. This is shown for the optimized procedure developed for the pyr. SiO_2_ powder sample (Table 11).

Finally, it was demonstrated that the application of an analytical technique that can be coupled directly to the robot-based station such as, e.g., AF4-MALS, enables a fully automatable analytical workflow from sample preparation to analysis. This thereby reduces human influence to a bare minimum. Such a setup not only helps save costly lab staff working hours but may eventually pave the way towards a fully automatable and standardizable workflow for the analysis of nanomaterials of different complexity thereby contributing to an improved comparability of data across different laboratories worldwide.

### 2.2. Automated Exposure of Cell Cultures at Air–Liquid Interface with the Possibility of Accompanied Physicochemical Investigations

In vitro methods for investigating inhalable nanomaterials are gaining increasing attention in scientific studies. Constant development of the techniques, cell models, and assays as well as the physiologically relevant exposure at the air–liquid interface enable profound insights into one of the relevant reception sites without using an entire organism [34,35].

**Figure 6:**
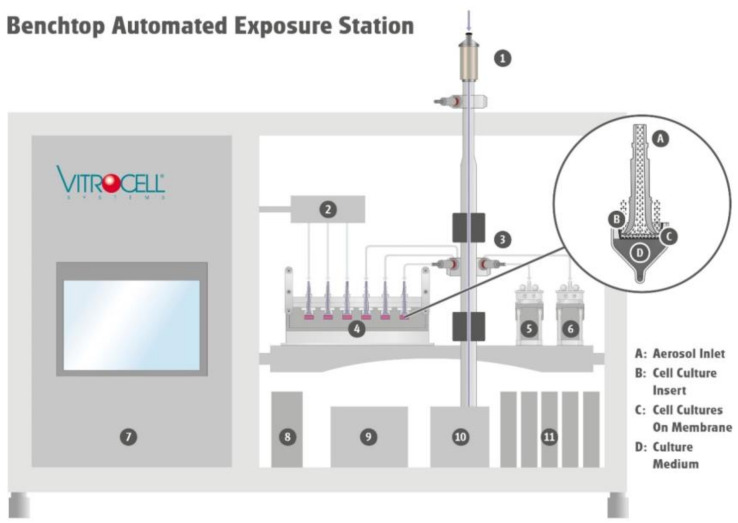
Schematic structure of the miniaturized Vitrocell Benchtop Automated Exposure Station. The aerosol is guided via the size-selective inlet (1) to the aerosol reactor and then conditioned to target temperature and humidity. It is further distributed through isokinetic sampling system (3) to the exposure modules (4–6), where cells are continuously exposed to the whole aerosol or clean air (2) at the air/liquid interface. During the exposure relevant parameters such as humidity of the aerosol reactor (8) and clean air control (2), cabinet temperature (9), and flows (11) are controlled (7). An integrated vacuum pump (10) provides vacuum at the respective flow controllers for aerosol reactor and exposure modules.

During the exposure at the air–liquid interface, monocultures or complex cell models are directly exposed to the whole aerosol, consisting of gas phase and particles (Figure 6). The aerosol enters the exposure module via the aerosol inlet and is guided over the lung cells. While the experiment is ongoing, cell cultures are supplied with culture medium from the bottom side via the porous membrane of the cell culture insert. Throughout the duration of an experiment, particles from the aerosol deposit on the cell surface and interact together with the gas phase. Excess aerosol is discharged back into the system via the aerosol outlet.

The necessary parameters for the exposure of cell cultures such as temperature, relative humidity of aerosol and clean air control, volume flow through the exposure module, test duration as well as start and end of an experiment can be controlled manually. All this requires a high level of user attention, particularly when an aerosol source needs to be controlled in parallel. If flows are not set correctly or positions of the exposure module are connected with a delay to the aerosol supply, non-reproducible test conditions arise that are difficult to trace back. In addition, cell cultures can show strong fluctuations and stable aerosol generation over several hours or even days, hence representing a major challenge in terms of reproducibility.

Therefore, automation and monitoring of the parameters are important steps for increasing the reproducibility and hence informative value of the data. Thus, the VITROCELL Automated Exposure Station was developed to convert the already established exposure at the air–liquid interface into an automated process [35]. With this system, important parameters of an experiment such as temperature, relative humidity, exposure duration, and volume flows can be defined in advance, are controlled to the desired target values and can be adjusted in real time during an experiment. For the duration of an experiment, parameters are automatically controlled, monitored, and recorded for later analysis. This allows a reproducible exposure of different monocultures as well as more complex cell models under realistic conditions with a subsequent comprehensive analysis [36,37,38].

Within ACEnano, the aim was to miniaturize the existing system into a benchtop version and further optimize it regarding automation and accompanying characterization. It was possible to reduce the dimensions of the existing systems from 2187 × 1124 × 623 to 700 × 1000 × 600 (height × width × depth in mm). With miniaturization, the number of positions for cell exposure changed, as well as the insert size from a 6-well to a 12-well format. There are now three positions for clean air control, three positions for aerosol exposure, and two additional positions for accompanying analytical measurements (see Figure 6).

We were able to demonstrate that the benchtop version works as reliably as the existing standard version and further showed a significant improvement in the humidification process (see Table 13).

With fluorescein sodium dosimetry described in [38], the deposited mass in every position of the exposure modules, served via the isokinetic sampling, was determined. As the surface of the 12-well insert as well as the flow rate is lower in comparison to the 6-well format, one would expect approximately four times less mass on the insert surface. The deposited mass of 102.37 ± 5.43 g^−9^ cm^−2^ h^−1^ correlates well with expected values. Further, the deviation within one experiment from each position of the exposure module is in the same range as of the standard version (standard version 5–8%, benchtop version 5–10%).

As already mentioned, an accompanying physicochemical characterization is essential for later evaluation of biological results and provides detailed insight into the physical properties and chemical composition of deposited particles on the cell surface. The analysis or sampling ideally takes place in separate analytical exposure modules but can also be integrated into normal exposure modules. For characterization, different methods are available to determine specific properties.

**Figure 7:**
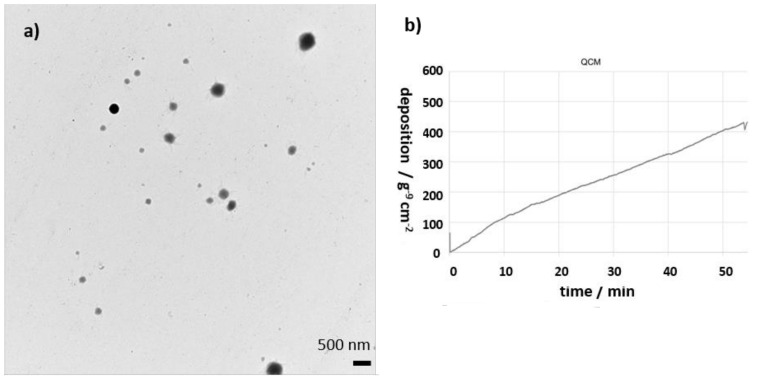
(**a**) An example of a TEM image of the fluorescein sodium aerosols from a grid which was exposed in a 12-well TEM insert in the second dosimetry module; (**b**) graph showing the deposited mass as a function of time on 12-well QCM within the Vitrocell 12/1 dosimetry module of the Benchtop Automated Exposure Station.

To enable reproducible physical characterization of the deposited particles, the existing TEM grid holder for the 6-well format of the original full-scale station [39] was further developed to fit into a 12-well format. The optimized design enables easier handling for the operator and a reproducible exposure of the TEM grids for every experiment. This new TEM grid holder is a fundamental analysis tool when it comes to aerosols, where the mean diameter of particles is smaller than 20 nm or the geometry of particles is of importance (e.g., fibers). An example of an image from an exposed TEM grid to fluorescein sodium aerosol is shown in Figure 7b. With an established cell preparation procedure after an experiment, confocal microscopy can be applied to investigate the interaction of particles and cells, e.g., particle translocation [40]. The deposited mass on the cell surface can be monitored online via an integrated quartz crystal microbalance (QCM, see Figure 7a).

However, there are limitations with QCM when deposited particles are very small (also depending on density and number concentration) or build out loose structures on the surface of QCM. For chemical characterization, an adapter can be mounted to the exposure module for direct sampling with a mass spectrometer. This allows the chemical characterization of exactly the same aerosol the cells are exposed to during the experiment.

The new control software of the benchtop version assists the user during operation and instructs how to proceed with the next steps of the process chain. The guidance ensures each step is carried out properly and therefore increases reproducibility. Previously defined protocols transfer target values of all relevant process parameters for each individual experiment to enable reproducible conditions for different experimental setups. 

However, with miniaturization and automation, new challenges arose. Due to miniaturization, new electronical components for the flow control, heating, illumination, pressure or humidity monitoring, and sensory integration were needed. Thus, the central control system faced a variety of different signals from analog to digital. Finding suitable components and sensors with compatible signal processing was one of the major challenges for the system automation to keep a central control system as small as possible. In the next step, a central control software has been designed and programmed to grant precise control of individual components to the right target values in defined time points.

Further development of an automated process for in vitro exposure at the air/liquid interface will lead to improved reproducibility and ease of use for this complex exposure technique. Secure and easy remote access to the system from outside of the lab is also of interest. It offers the operator a simple way to check the experiment frequently without being forced to stay in the proximity of the system during the whole duration of the experiment.

## 3. Discussion

Reproducibility of results will only be achieved when appropriate procedures and protocols are accepted and applied by the majority of the community. Standards are a useful tool to harmonize such procedures. For the sample preparation of nanomaterials, the first steps have been carried out, but in comparison to the standardization of measurements there is still a long way to go. The automation of proper measurement procedures simplifies the use of the equipment and boosts the standardization and reproducibility. Nowadays, there are no problems reproducing the results of identical samples on different instruments. For modern apparatuses, the measurement conditions are recorded and saved in the metadata automatically. As a result, the main reason for varying results is the different sample preparation. Although the latter statement is broadly accepted and sample preparation is considered in the cause-and-effect analyses, systematic studies are still missing [41,42]. An actual interlaboratory comparison is ongoing to investigate the effect of different preparation methods systematically for ToF-SIMS [43]. 

The aim of this publication is to discuss how and when automated preparation can enhance the quality of the measurement results. The first comparison between manual and automatized sample preparation presented in Section 2.1. highlighted similar results. It is reasonable to set the benchmark for automated protocols to the comparable manual procedures performed by an experienced experimentalist. Then, the necessary investment for the equipment can be balanced with saving costly working hours. Furthermore, time-consuming systematic optimization of preparation protocols evaluating different working conditions such as temperature, environment, humidity, mixing, and statistical evaluations to obtain more insights into the quantitative uncertainties of the measurements can be obtained without needing a great amount of working hours of expensive and highly skilled experimentalists.

In terms of reproducibility, digital standard operating procedures and an automated recording of the preparation conditions are great advantages. Only by means of this approach can human bias correlated with the skill and the experience of the experimentalist be excluded [2]. The digital operating protocols with the exact preparation conditions should belong to the metadata of the measurement results such as the measurement conditions. Only then can the reproducibility of the analytical results of the same materials be ensured in different labs. In other words, measured differences can be correlated to the sample, and any preparation effects can be excluded. With such an approach, data with the necessary metadata from different laboratories can be used allowing to achieve the necessary amount of data for machine learning and artificial intelligence. Only through such an approach can the new possibilities of data handling be exploited for their strength. It is expected that new reliable structure–activity relationships can be established with such novel approaches which are rarely found associated with traditional methods.

Another experimental approach to establish structure–activity relationships is correlative measurements combining different analytical methods. Therefore, an air–liquid interface which is presented in Section 2.2, was developed. This example showed that automation is possible for rather complex samples. Biological samples can be prepared in a reproducible manner, under exactly the same conditions on a TEM grid for the analysis of size and shape. Furthermore, chemical analysis can be performed by means of mass spectrometry.

For the majority of analytical methods in nano-analytics, automated preparation methods are available, but they are not popular. Their cost is rather high, but valuable working time can be saved or used for non-routine tasks as shown in example Section 2.1. Furthermore, coupling the preparation equipment with analytical tools or combining the preparation of biological samples with physicochemical methods allows new approaches for reliable correlative measurements. This is shown in Section 2.2. and is necessary for quantitative structure–activity relationships and modern methods in risk assessment [27]. On the other hand, automation is not feasible for sample preparation methods which are rarely used or require long experimental experience.

The use of automatic digital standard operating procedures and the recording of all variables will especially influence the analytical results and hence, are inherent. For defining these variables, a suitable cause-and-effect analysis with a critical view of the quantitative uncertainties is necessary. Such cause-and-effect analysis is a conceptual process that can help to guide robustness testing and determine process control measures that should be included in a protocol to support charting of important sources of variability. It is easy to realize such control measures in an automated system with process control. With such procedures, the reproducibility and the reliability of the results should be ensured. Recently, an air–liquid exposure system was optimized for the uptake of CeO_2_ nanoparticles to resemble in vivo intracellular nanoparticle deposition, observed in an animal study using female Wistar rats [44]. The optimization was achieved using a cause-and-effect analysis approach to monitor cell viability within the exposure system and pinpoint the key sources of experimental variability [41,45,46,47]. This study unambiguously showed that the air–liquid exposure system can be standardized to resemble in vitro inhalation study exposure data. Furthermore, it can be used within this framework within a regulatory context where standardized and validated in vitro methods are needed to actually resemble in vivo inhalation studies and therefore make a major contribution to the 3R (reduction, replacement, and refinement) paradigm to replace animal in vivo studies using hyphenated in vitro exposure experiments.

A good example of the advantage of automation is bio-analytics. In recent years, promising developments could be observed which show the advantages of automation. A great number of complex samples and the need for fast analysis require the development of high-throughput techniques; on the other hand, reliable and precise measurements are crucial in clinical practice or in medical diagnostic, e.g., in cancer screening. Therefore, great efforts were made to integrate multiple laboratory steps, minimize sample consumption, and increase the analysis speed and data precision which can be realized by microfluidic technology [48]. Establishing new protocols using tailored nanomaterials can help to minimize the preparation due to the enhancement in the detection [49,50,51,52,53]. Furthermore, the use of nanomaterials in such important fields stresses the significance of fast and reliable nano-analytics. Minimization of the analytical equipment with lab-on-a-chip technology enables fast, sensitive, and selective detection and minimizes the amount of needed sample material [53]. Machine learning allows computer-aided fingerprinting and multi-modal recognition integrating constraints for a fast screening of the samples [50,51,52,53,54].

All these recently developed bio-analytic systems show in an exemplary way that the analytical workflow (Figure 1) must be viewed in unity and lead the way for the development of automated nano-analytics which will become essential in the immediate future. Strong cooperation between experts in different fields of analytics including experimentalists, instrument developers, software experts, and experts in standardization will pave the way for success.

To conclude, in addition to the automation of measurements which is reasonably well established, the automation of sample preparation is an important step in producing reliable data which is necessary for modern data analytical tools such as machine learning. Thereby, the reduction in manual input can help obtain the amount of data necessary for a rigorous statistical evaluation without the need for repetitive human activities; in turn, minimizing the human bias of sample preparation which is often not a predictable source of uncertainty.

## Figures and Tables

**Table 1:** Methods appropriate for nanoparticle characterization in the form of suspension. In this table, as well as in the subsequent Table 2, Table 3 and Table 4, the properties measured with each specific method are mentioned. The generic challenges and advantages of each group of sample preparation methods are presented.

Preparation	Analytical Method	Properties Measured	Challenges	Advantages
Suspension	FFF (field-flow fractionation)	Particle size following particle fractionation	Prevention of agglomeration/sedimentation, suitable concentration	Easy, in situ or operando analytics possible
	HDC (hydrodynamic chromatography)	Particle size following particle fractionation		
	SP-ICP-MS (single particle ICP-MS)	Mass-based particle size, mass-based size distribution, number concentration, compositional heterogeneity of the particles		
	SEC (size exclusion chromatography), HPLC (high-performance liquid chromatography)	Particle size (hydrodynamic volume)		
	HIC (hydrophobic interaction chromatography)	Hydrophobicity		
	PTA (particle tracking analysis)	Hydrodynamic particle size and distribution, number concentration		
	SAXS (small angle X-ray scattering)	Particle size distribution		
	DLS (dynamic light scattering)	Particle size, zeta potential (for instruments with electrophoretic light scattering)		
	CE (capillary electrophoresis)	Separation NMs of varying size, shapes, surface modifications and composition		

**Table 2:** Methods appropriate for nanoparticle characterization in dry sample form. The column headings are explained in the caption of Table 1.

Preparation	Analytical Method	Properties Measured	Challenges	Advantages
Dried suspension (e.g., drop-cast, spin-coated)	XPS (X-ray photoelectron spectroscopy)	Surface chemistry, composition	Prevention of agglomeration, homogeneous and gapless coating, stable fixation, prevention of contamination, sample alteration, loss of materials (spin-coating), dependent on substrate quality	Secure fixation, consistency of results (spin-coat), well established reproducible methods
	SIMS (secondary ion mass spectrometry)	Surface chemistry		
	TEM (transmission electron microscopy)	Particle primary size and shape		
	SEM (scanning electron microscopy)	Particle primary size and morphology		
	EDS (energy-dispersive spectroscopy)	Bulk composition		
	AES (Auger electron spectroscopy)	Composition of the surface		
	AFM (atomic force microscopy)	Particle size, morphology		
	STXM (scanning transmission X-ray microscopy)	Particle primary size, shape composition, and chemistry. chemical heterogeneity of the particles		
	ATR-FT-IR (attenuation total reflection Fourier-transform infrared) spectroscopy	Surface chemistry, chemical composition		

**Table 3:** Methods appropriate for nanoparticle characterization in powder form. The column headings are explained in the caption of Table 1.

Preparation	Analytical Method	Properties Measured	Challenges	Advantages
powder	XPS (X-ray photoelectron spectroscopy)	Surface chemistry, composition	Stable fixation, prevention of agglomeration, prevention of contamination	Little sample preparation required, maintains integrity of the sample
	SIMS (secondary ion mass spectrometry)	Surface chemistry, composition		
	TEM (transmission electron microscopy)	Particle size and shape		
	SEM (scanning electron microscopy)	Particle size and morphology		
	EDS (energy-dispersive spectroscopy)	Composition		
	AES (Auger electron spectroscopy)	Composition of the surface		
	BET (Brunauer–Emmett–Teller)	Surface Area, porosity, pore distribution		
	TGA (thermo gravimetry analysis)	Weight loss during thermal decomposition of the sample		
	STXM (scanning transmission X-ray microscopy)	Particle size, shape, composition, and chemistry		
	SAXS (small-angle X-ray scattering)	Particle size and distribution		

**Table 4:** Methods appropriate for nanoparticle characterization in the form of pellets. The column headings are explained in the caption of Table 1.

Preparation	Analytical Method	Properties Measured	Challenges	Advantages
Pellet	XPS (X-ray photoelectron spectroscopy)	Surface chemistry, composition	Prevention of contamination, danger to integrity of the sample (both surface and shape)	Secure fixation
	SIMS (secondary ion mass spectrometry)	Surface chemistry		
	FT-IR (Fourier-transform infrared spectroscopy)	Surface chemistry, chemical composition		

**Table 5:** Methods appropriate for nanoparticle characterization involving less-common pre-analysis treatment. The column headings are explained in the caption of Table 1.

Preparation	Analytical Method	Challenges	Advantages
Cryo treatment	Cryo fixation for XPS and ToF-SIMS, freeze drying for XPS, TG, SEM, TEM	Prevention of crystallization, experimental experience is required, costs	Integrity of the surrounding (biological media)
Microprinting	ToF-SIMS, AES, TEM, SEM	Particle density, coffee-ring effects	Easy-to-handle, high automation potential
Fixation on or embedding in a (polymer) matrix	ToF-SIMS, TEM, SEM	Experimental experience is required, suitable matrix, reduction of sample integrity	Single particle imaging or mapping
Electrospray deposition	TEM, SEM	Expensive equipment, aqueous solution	Quantitative, useful for depositing magnetic NPs

**Table 6:** Selection of international standards concerning sample preparation. This list presents a range of standards but does not claim to be exhaustive. More information on each standard listed below is shown in Supplementary Materials Table S1.

Standard	Title
ISO TR 20489:2018	Nanotechnologies—Sample preparation for the characterization of metal and metal-oxide nano-objects in water samples
	data
ISO TR 19716:2016	Nanotechnologies—Characterization of cellulose nanocrystals
ISO TS 21346:2021	Nanotechnologies—Characterization of individualized cellulose nanofibril samples
ISO TS 21356:2021	Nanotechnologies—Structural characterization of graphene–part 1: graphene from powders and dispersion
ISO 20579-4:2018	Surface chemical analysis—Guidelines to sample handling, preparation and mounting–part 4: reporting information related to the history, preparation, handling and mounting of nano-objects prior to surface analysis
CEN TS 17273	Nanotechnologies—Guidance on detection and identification of nano-objects in complex matrices

**Table 7:** Detailed information about the nanomaterial samples subjected to both manual and automated sample preparation using the robot-based station.

Sample Name	Gold Nanoparticles	Polyvinylpyrrolidone-Coated Titania Nanoparticles	Pyrogenic Silica Particles, HDK^®^ D05
Abbreviation	AuNP	TiO_2_-PVP	pyr. SiO_2_
Supplier	BBI Solutions, UK	Promethean Particles, UK	Wacker Chemie, DE
Size/dispersity	60 nm/monodisperse	12 nm primary particle/polydisperse	174 nm/polydisperse
Physical state	Suspension	Suspension	Powder
Initial mass concentration	5 mg/L, suspended in 0.2% NovaChem *	500 mg/L, suspended in 0.2% NovaChem *	Not applicable
Stability of suspension	Stable	Moderate	Not applicable

* NovaChem is a mixture of different salts and surfactants commercially available from Postnova Analytics GmbH.

**Table 8:** Applied protocols for the sample preparation procedure performed both using the robot-based station and manually by experienced lab staff.

Sample Preparation Procedure	AuNP	TiO_2_-PVP	Pyr. SiO_2_
Suspending	Not applicable	Not applicable	10 mg powder in 4 mL 0.1 mM aqueous KOH, 2.5 mg/mL
Diluting	1:2.5; 1:5; 1:8.3; 1:25 in 0.2% NovaChem (*v/v*)	1:6; 1:10; 1:30 in 0.2% NovaChem (*v/v*)	1:6; 1:10; 1:30 in 0.1 mM aqueous KOH (*v/v*)
Vortexing	1 min per sample	Not applied	Not applied
Mixing	Shaking by hand or “Draw&Dispense” function	Shaking by hand or “Draw&Dispense” function	Shaking by hand or “Draw&Dispense” function
Ultrasonication	Not applied	3 × 3 min (pulsed: amplitude 100% with 70% power on and 30% power off	3 × 3 min (pulsed: amplitude 100% with 70% power on and 30% power off

**Table 9:** Squared correlation coefficients R^2^ obtained from UV absorption measurements (Postnova PN3211 UV detector, 254 nm detection wavelength) of a dilution series of the three investigated nanomaterial samples according to the automated and manual approach described in Table 8 (line “Diluting”). Standard deviation (SD) is calculated from the arithmetic mean of the UV absorption values obtained for all dilution ratios normalized to the initial nanoparticle mass concentration with each prepared sample aliquot measured in triplicate.

Linearity of Dilution	AuNP Automated Manual		TiO_2_-PVP Automated Manual		Pyr. SiO_2_ Automated Manual	
R^2^	0.9998	0.9999	0.9994	0.9998	0.9983	0.9996
SD (%)	<0.1	<0.1	<0.1	<0.1	<0.5	<0.1

**Table 10:** Hydrodynamic diameter (D_h_, z-average, Malvern Zetasizer Nano ZS, cumulant analysis) of the TiO_2_-PVP and pyr. SiO_2_ sample processed both automatically and manually according to the procedure described in Table 8. Standard deviation (SD) is calculated from the arithmetic mean of three independent DLS measurements performed on each of the six sample aliquots.

Size	TiO_2_-PVP Automated Manual		Pyr. SiO_2_ Automated Manual	
D_h, z-average_ (nm)	116.6	118.7	378.3	310.0
SD (%)	4.7	6.3	17.9	8.9

**Table 11:** Applied optimized protocol for the sample preparation procedure for the pyr. SiO_2_ powder performed both automatically using the robot-based station and manually by experienced lab staff.

Optimized Sample Preparation Procedure	Pyr. SiO_2_
Suspending	6 mg powder in 1.5 mL 0.2% NovaChem, 4.0 mg/mL
Mixing	Shaking by hand or “Draw&Dispense” function
Ultrasonication	1 × 3 min (pulsed: amplitude 100% with 70% power on and 30% power off)
Dilution	1:2 in 0.2% NovaChem, 2.0 mg/mL (*v/v*)
Mixing	Shaking by hand or “Draw&Dispense” function
Ultrasonication	2 × 3 min (pulsed: amplitude 100% with 70% power on and 30% power off)
Dilution	1:20 in 0.2% NovaChem, 100.0 mg/mL (*v/v*)
Mixing	Shaking by hand or “Draw&Dispense” function
Ultrasonication	3 × 3 min (pulsed: amplitude 100% with 70% power on and 30% power off)

**Table 12:** Summary of the AF4-MALS results obtained for six pyr. SiO_2_ samples that were prepared independently either automatically by the robot-based station or manually by experienced lab staff following the procedure described in Table 11. Displayed errors represent the calculated standard deviation (SD) in percent obtained from the mean of single measurements of six independent samples.

AF4-MALS Results	Automated Preparation	Manual Preparation	Deviation
Dg at 90° MALS signal maximum (nm)	378.5 ± 2.4%	406.6 ± 1.9%	6.9%
Arithmetic mean Dg from 18–32.5 min (nm)	502.4 ± 2.8%	529.3 ± 3.7%	5.1%
Full width at half maximum, FWHM, 90° MALS signal (min)	11.6 ± 6.7%	12.4 ± 3.0%	6.5%

**Table 13:** Comparison of the most important control variables and parameters of the two different systems.

	Standard Version		Benchtop Version	
	Mean	Deviation	Mean	Deviation
Temperature cabinet Set point 37 °C	36.99	+/−0.03	36.61	+/−0.03
Humidity aerosol reactor Set point 85% r.h.	84.79	+/−1.33	85.02	+/−0.13
Humidity clean air control Set point 85% r.h.	84.78	+/−0.81	85.00	+/−0.12
Deposited mass in g^−9^ cm^−2^ h^−1^)	290.00	+/−37.50	102.37	+/−5.43
Dimensions [height × with × depth in mm]	2187 × 1124 × 623		700 × 1000 × 600	

## Data Availability

Data can be made available upon request.

## Supplementary Materials

The following are available online, S.1: Microprinting of nanoparticles for electron microscopy analysis, S.2: Standards concerning sample preparation and links, S.3: Conditions of the AF4-MALS analysis.

## Sample Availability

Samples of the compounds are not available from the authors.
