# Supplementary material for: Automation and Standardization—A Coupled Approach towards Reproducible Sample Preparation Protocols for Nanomaterial Analysis
Source: Molecules. 2022 Feb 1;27(3):985. doi: 10.3390/molecules27030985 (PMC8838799; doi:10.3390/molecules27030985)
Supplement: Supplementary file 1 [file molecules-27-00985-s001.zip › molecules-1560739-supplementary.pdf]

Supporting information

Automation and Standardization – a coupled approach towards reproducible sample preparation protocols for nanomaterial analysis

Jörg Radnik [1,*], Vasile-Dan Hodoroaba [1], Harald Jungnickel [2], Jutta Tentschert [2], Andreas Luch [2], Vanessa Sogne [3], Florian Meier [3], Loïc Burr [4], David Schmid [4], Christoph Schlager [5], Tae-Hyun Yoon [6,7], Ruud Peters [8], Sophie M. Briffa [9], and Eugenia Valsami-Jones[9]

S.1. Microprinting of nanoparticles for electron microscopy analysis

The principle of the developed technique is to print nano- or pico-droplets of nanomaterial suspensions on substrates such as TEM grids. For this, Cu TEM grids deposited on clean microscopy glass slides were printed with a 4x4 array of 0.35-0.4 nL droplets, using a NanoPlotter 2.0 (GeSIM GmbH) piezo-electric printer equipped with NanoTips J piezoelectrical pipette tips (Figure S1). As part of the ACEnano project, focus was set on particles already in suspension, whereas an additional step to suspend nanomaterials would also allow the use of powder samples.

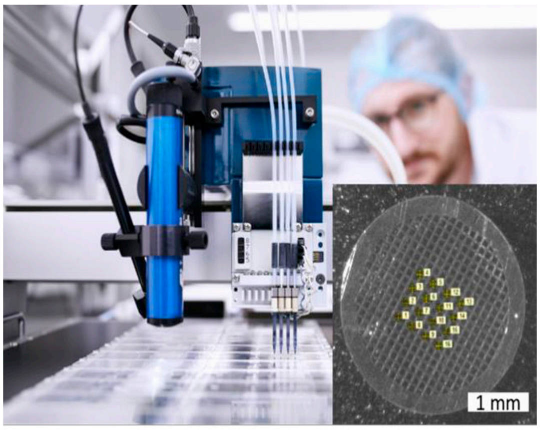

Figure S1: Picture of the Nanoplotter device at CSEM equipped with 4 piezoelectric pipette tips and microscopy image of the TEM grid with overlapped 4x4 printing array.

To enable the acquisition of useful EM images, several requirements must be met. As for the standard EM preparation method, impurities must be removed from the nanomaterial samples so as to not dominate the acquired image. For this cleaning procedures similar to standard sample preparation techniques such as dialysis or centrifugation/re-suspension can be used. On the one hand nanomaterials must form no more than a monolayer on the substrate so that each nanoobject can be individually identified and quantified. On the other hand, nanomaterial density as deposited must be sufficient to enable efficient data acquisition of a statistically relevant number of nanomaterials qualitative and quantitative characterization.

While the automation of dispensing nanomaterials on suitable substrates present major advantages such as high throughput and quantitative measurements, it gives rise to new challenges. Agglomeration and aggregation in suspension must be prevented to avoid clogging the piezoelectrical pipette tips. More importantly, the drying of the printed droplets must be controlled to avoid formation of agglomerates / aggregates and “coffee rings”. However, as for standard methods, cleaning procedures will involve similar bias to the printed sample. In the present work, to minimize alteration of the

nanomaterials, particle suspensions were only modified by dilution with ultrapure water. Droplet drying has been tuned by varying temperature and humidity. Figure S2 shows STEM-in-SEM images of a single microprinted droplet of 100 nm latex NPs. It evidences that printing at a concentration of 4.5 x 1010 nanoparticles per ml and drying at 21 °C with 55% relative humidity led to the formation of a printed spot of few tenths of micrometre in diameter with a "coffee-ring" with high particle density and inner surfacee with low particle density.

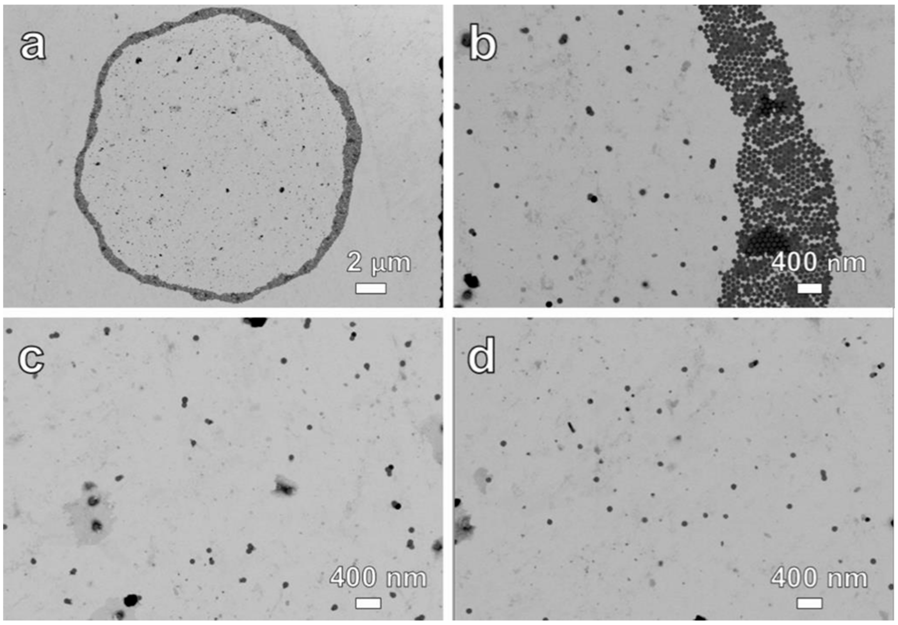

Figure S2: Zeiss Supra 40 high magnification T-SEM micrographs of a micro-printed 100 nm latex nanoparticles at concentration of 4.5 x 1010 NP/mL. a) Low magnification micrograph showing an entire 400 pL droplet after microprinting, b) magnification on the edge of the printed droplet evidencing particle agglogmeration in the shape of a"coffee ring", c) and d): magnification in the center of the droplet showing mostly individual particles homogeneously distributed with low density.

During the ACEnano project, the development of the microprinting of nanoparticles made the present technique very promising for increasing the use of imaging as a standard analysis method. However, several challenges remain for its broad application. Further optimisation of the particle concentration in suspension and optimisation of the droplet drying process on the substrate are necessary to avoid agglomerates and coffee rings as shown in Figure S2. In addition, improvement of the alignment technique using computer assisted detection of alignment markers would increase precision and speed of printing. Further improvements would involve printing of even smaller droplets, which in turn would enable faster imaging of entire drops and the printing of 1 drop per TEM grid cell leading to more than 100 different samples on one single TEM grid.

S.2 Standards concerning sample preparation and links

Standard	Title	Link / summara

ISO TR 20489:2018	Nanotechnologies – Sample preparation for the characterization of metal and metal-oxide nano-objects in water samples	https://www.iso.org/standard/68198.html<br><br>Sample preparation (i.e. pretreatment and size-fractionation) for analytical measurements applied to surface and drinking water containing relevant amounts of metal and metal oxides
ISO TR 19716:2016	Nanotechnologies – Characterization of cellulose nanocrystals	https://www.iso.org/standard/66110.html<br><br>Methods for the characterization of cellulose nanocrystals including sample preparation, measurements methods and data analysis
ISO TS 21346:2021	Nanotechnologies – Compilation and description of sample preparation and dosing methods for engineered and manufactured nanomaterials	https://www.iso.org/standard/70638.html<br><br>Characteristics to be measured of individualized cellulose nanofibril in suspension and powder form and their measurement methods, including sample preparation, measurement and data analysis procedures
ISO TS 21356:2021	Nanotechnologies – Structural characterization of graphene – part 1: graphene from powders and dispersion	https://www.iso.org/standard/70757.html<br><br>Sequence of methods for the characterization of structural properties of graphene, bilayer graphene, and graphene nanoplatelets from powders and liquid dispersions. A range of measurement techniques is presented after the isolation of individual flakes on a substrate which provided properties like thickness, lateral flake size, level of disorder, layer alignment and specific surface area. Measurement protocols, sample preparation and data analysis routines are provided
ISO 20579-4:2018	Surface chemical analysis – Guidelines to sample handling, preparation and mounting – part 4: reporting information related to the history, preparation, handling and mounting of nano-objects prior to surface analysis	https://www.iso.org/standard/68833.html<br><br>Information is identified to be reported in a datasheet, certificate of analysis, report or other publication regarding the handling of nano-objects in preparation for surface chemical analysis
CEN TS 17273	Nanotechnologies – Guidance on detection and identification of nano-objects in complex matrices	https://www.en-standard.eu/pd-cen-ts-17273-2018-nanotechnologies-guidance-on-detection-and-identification-of-nano-objects-in-complex-matrices/<br><br>Requirements for sampling and treatment of complex matrices like liquid environmental compartments, waste water and consumer products in order to obtain a liquid dispersion with sufficiently high concentration of the nano-objects of interest. The selected analysis methods are based on a combination of size classification and chemical composition analysis (FFF, EM and sp-ICP-MS).

S.3. Conditions of the AF4-MALS analysis

Sample information

Sample: pyr. SiO_2 suspension, 100 mg/L suspended in eluent after manual and automated preparation Injection volume: 100 µL (10 µg injected mass)
Eluent: 0.2 % NovaChem

AF4-MALS setup

AF4: Postnova AF2000 MultiFlow equipped with a Postnova PN1650 Smart Stream Splitting Module MALS: Postnova PN3621 MALS

- 532 nm Laser
- 80% Laser power
- data fitting via random coil model, 19 active angles (12°-156°)

AF4-fractionation conditions

Sample introduction

- 0.2 mL/min injection flow
- 2 min delay time
- 7 min injection time
- 0.5 min transition time

Flow conditions

- Cross flow profile (see Figure S3)
    - 1.5 mL/min initial cross flow field
    - 0.2 min constant cross flow field
    - 10 min power decay cross flow field (exponent 0.1) to 0.1 mL/min
    - 35 min constant cross flow field at 0.1 mL/min
- 0.5 mL/min channel flow
- 50% slot-outlet

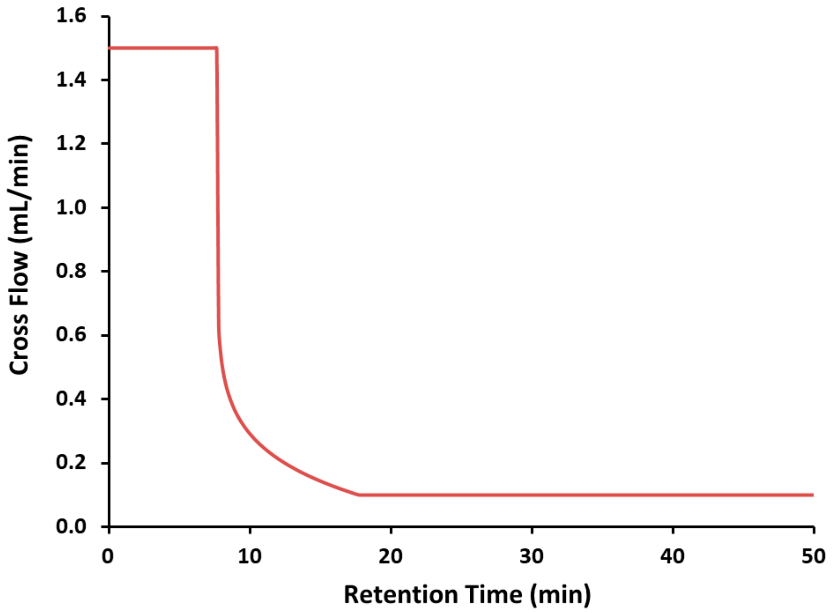

Figure S3: Cross flow profile that was used for the fractionations
